# Exploring associations between personality trait facets and emotional, psychological and social well-being in eating disorder patients

**DOI:** 10.1007/s40519-021-01107-6

**Published:** 2021-03-09

**Authors:** Jan Alexander de Vos, Mirjam Radstaak, Ernst T. Bohlmeijer, Gerben J. Westerhof

**Affiliations:** 1 University of Twente, Department of Psychology, Health, and Technology, Enschede, The Netherlands; 2 Stichting Human Concern, Centrum voor Eetstoornissen, Amsterdam, The Netherlands; 3 Centre for eHealth and Well-Being Research, Enschede, The Netherlands

## Abstract

**Purpose:**

Personality functioning is strongly linked to well-being in the general population. Yet, there is a lack of scientific knowledge about the pathways between personality trait facets and emotional, psychological and social well-being in ED patients. The general aim was to examine potential associations between maladaptive personality trait facets and the three main dimensions of well-being.

**Methods:**

Participants were 1187 female eating disorder patients who were referred for specialized treatment. Patients were diagnosed with anorexia nervosa (31.7%), bulimia nervosa (21.7%), binge eating disorder (11%) and other specified eating disorders (35.5%). The Personality Inventory for the DSM 5 (PID-5) was used to measure 25 trait facets, and well-being was measured with the Mental Health Continuum Short Form (MHC-SF). Multiple hierarchical regression analyses were applied to examine potential associations between personality and well-being while controlling for background and illness characteristics.

**Results:**

Personality trait facets led to a statistically significant increase of the explained variance in emotional (38%), psychological (39%), and social well-being (26%) in addition to the background and illness characteristics. The personality trait facets anhedonia and depression were strongly associated with all three well-being dimensions.

**Conclusion:**

Personality traits may play an essential role in the experience of well-being among patients with EDs. To promote overall mental health, it may be critical for clinicians to address relevant personality trait facets, such as anhedonia and depression, associated with well-being in treatment.

**Level of evidence:**

Level V, cross-sectional descriptive study.

**Supplementary Information:**

The online version contains supplementary material available at 10.1007/s40519-021-01107-6.

## Introduction

Personality trait facets are relatively stable patterns of behaviors, cognitions, and emotions, which develop during childhood and adolescence. Traits can be placed on a continuum from normality to pathology (dimensional), which means that they can develop in a healthy way or become maladaptive [1].

Decades of research highlight that personality plays a critical role in how people approach and appraise their lives and experience well-being [2]. Personality traits are strongly linked with the experience of subjective and psychological well-being (PWB) in the general population [2]. Subjective well-being, also described as emotional well-being (EWB), consists of three dimensions, life satisfaction, positive, and negative affect [3]. PWB or Eudaimonic well-being is about living a good life and is conceptualized in six dimensions; self-acceptance, positive relationships, autonomy, environmental mastery, personal growth, and purpose in life [3]. Social well-being (SWB) has been proposed as a third main factor of well-being, consisting of five dimensions, actualization, coherence, integration, contribution, and acceptance [3]. Well-being and psychopathology are considered as two related, but distinct dimensions of mental health, with potentially different determinants [4].

In a comprehensive meta-analysis, it was found that on a domain level, neuroticism, extraversion and conscientiousness were strongly associated with EWB and PWB [2]. However, analysis on a trait facet level (i.e., a specific and unique aspect of a broader personality domain) provided a more detailed description of the relationships between personality and well-being and improved the incremental prediction with 20% [2]. Multiple personality trait facets, such as anxiety, hostility, depression, self-consciousness, vulnerability, warmth, assertiveness, positive emotions, trust, competence, achievement striving, and self-discipline, were moderately to strongly associated with EWB and PWB [2].

Studies examining personality functioning in patients with eating disorders have primarily focused on explaining ED pathology [5]. Personality is strongly linked to the onset and maintenance of eating disorders (EDs), in particular perfectionism, neuroticism (i.e., depression, anxiety, anhedonia, impulsiveness, and stress vulnerability), avoidance motivation, sensitivity (to social rewards), extraversion, and self-directedness [5].

Knowledge concerning the role of personality functioning for the experience of well-being in ED patients is sparse, while they experience lower levels compared to the general population [6]. Available studies have focused primarily on specific aspects of personality such as resilience, or specific domains of Quality of Life (QoL) [7]. Although some QoL domains have conceptual overlap with dimensions of well-being (e.g., emotional and social functioning), PWB is often neglected as a domain in QoL measures. Yet, PWB has a strong theoretical background, dating back to Greek philosophy, and is considered as one of the most influential models of mental health [2, 3].

Well-being is also important to consider as a measure for recovery in addition to symptom remission because people who have recovered from an ED, consider the presence of well-being essential for recovery [6]. Since personality trait facets are strongly linked to the onset and maintenance of EDs, and the experience of well-being in the general populations, it may function as an underlying maintaining factor for overall mental health (i.e., the presence of well-being and low levels of psychopathology). To improve well-being, as well as reduce ED pathology among patients, it may therefore be crucial to promote the strengthening of adaptive personality trait facets in treatment. Examining which specific personality trait facets are linked to the dimensions of well-being in ED patients may provide clinicians with knowledge on which trait facets to focus on in treatment.

This study, therefore, aimed to examine potential associations between maladaptive personality trait facets and the three main dimensions of well-being (emotional, psychological, and social) on a trait facet level in a transdiagnostic ED sample of patients with anorexia nervosa (AN), bulimia nervosa (BN) binge eating disorders (BED), and other specified feeding and eating disorders (OSFED).

## Methods

### Participants and procedure

Participants were Dutch ED patients, referred for treatment at Stichting Human Concern, a specialized centre for the treatment of EDs. General practitioners referred patients to specialized care with a reference for further diagnosis or treatment. The inclusion criteria were: (1) a minimum age of 17 years, (2) a primary ED diagnosis at intake, achieved according to the criteria of the diagnostic and statistical manual (DSM-5), (3) being able to understand and fill in the questionnaires, and (4) consent to participate in the research. A total of 1356 patients were screened between January 2016 and March 2020 and received a written brochure about the aim of the study and options for contacting the researchers. Informed consent included that participants were informed about the study and could withdraw their data for scientific research at any time. The Behavioral, Management and Social Sciences Ethics committee of the University of Twente approved the study protocol. One hundred and thirty-two patients did not give consent, and 37 men were excluded leading to a total of 1187 included patients. Patients were diagnosed by a psychiatrist in collaboration with an intake team, consisting of a family therapist, dietician, and a psychologist.

### Data collection

The following background and illness characteristics were collected during intake: age (*M* = 26.9 years, *SD* = 8.9, range 17–66), start age ED (16.5 years, *SD* = 5.6, range 4–55), ED duration (9.6 years, *SD* = 8.9, range 0.25–49), BMI kg/m^2^ (M = 22.5, *SD *= 7.4, range 10.2–59), ED diagnosis (32.1% AN, 22.2% BN, 11% BED and 34.7% OSFED) and having a comorbid personality disorder (11%), or other psychiatric disorder (50.8%). Other psychiatric disorders were, mood and anxiety, developmental, trauma-related, neurocognitive, and addictive disorders.

Personality trait facets were measured with the Dutch self-report Personality Inventory for DSM 5 (PID-5) [8] according to the dimensional model of personality [1]. The PID-5 is a 220-item self-report measure, designed to assess personality domains (antagonism, detachment, disinhibition, negative affectivity, and psychoticism) and 25 underlying trait facets, included in the DSM-5 alternative dimensional model [8]. The items are evaluated on a 4-point Likert scale, ranging from 0 (very false or often false) to 3 (very true or often true). An example question from the personality trait facet impulsivity is “I always do things on the spur of the moment”. Higher scores are indicative of higher maladaptive personality functioning. The internal consistencies were acceptable to excellent with excellent mean inter-item correlations (see Table 1).

**Table 1:** Mean scores of the PID-5 personality trait facets and scale statistics

Trait facet	*N* questions	*M* (*SD*)	Cronbach’s alpha (α)	Mean inter-item correlations
Anhedonia	8	1.26 (.60)	.83	.37
Anxiousness	9	1.58 (.62)	.85	.38
Attention seeking	8	.78 (.60)	.86	.44
Callousness	14	.19 (.23)	.75	.23
Deceitfulness	10	.47 (.43)	.82	.34
Depression	14	1.35 (.67)	.92	.44
Distractibility	9	1.50 (.72)	.90	.50
Eccentricity	13	.81 (.63)	.92	.48
Emotional lability	7	1.66 (.69)	.87	.48
Grandiosity	6	.28 (.35)	.69	.30
Hostility	10	.87 (.52)	.83	.33
Impulsivity	6	.83 (.67)	.89	.57
Intimacy avoidance	6	.82 (.72)	.84	.48
Irresponsibility	7	.56 (.50)	.77	.33
Manipulativeness	5	.52 (.50)	.72	.34
Percept. dysreg	12	.63 (.46)	.78	.26
Perseveration	9	1.45 (.60)	.82	.33
Restricted affec	7	.97 (.62)	.81	.39
Rigid perfectionism	10	1.52 (.68)	.88	.43
Risk taking	14	1.15 (.50)	.87	.32
Separation ins	7	1.12 (.64)	.80	.36
Submissiveness	4	1.65 (.75)	.86	.60
Suspiciousness	7	1.00 (.57)	.78	.35
UBE	8	.37 (.44)	.79	.33
Withdrawal	10	1.00 (.62)	.90	.46

*Percept. Dysreg.* perceptual dysregulation, *Restricted affect.* restricted affectivity, *Separation ins.* separation insecurity, *UBE* unusual beliefs and expectations

Well-being was measured with the Dutch Mental Health Continuum Short Form (MHC-SF) [3]. The MHC-SF consists of 14 items and measures emotional (*N* = 3), psychological (*N* = 6), social (*N* = 4), and overall well-being. The items are rated on a 6-point Likert scale ranging from 0 “never” to 5 “always,” and an example question from the dimension PWB is “During the past month, how often did you feel that you had warm and trusting relationships”. Higher scores are indicative of higher levels of well-being. The internal consistency of the scales was 0.83 for emotional, 0.82 for psychological and 0.72 for social well-being.

The Dutch 36 item Eating Disorder Examination (EDE-Q) was used to measure ED psychopathology (EDP) with the global score [9]. The internal consistency of the global scale was 0.92.

### Analysis

Three multiple hierarchical regression analyses were run with emotional, psychological, and social well-being as dependent variables and the background and illness characteristics and personality trait facets as independent variables. A hierarchical model was tested in two steps and compared on model fit, explained variance and stability of the associations. In step 1, the background and illness characteristics were entered, and in step 2, personality trait facets were added. The assumptions for linearity, homoscedasticity, and normality were met as inspected with QQ plots and histograms. There was no multicollinearity between the independent variables as inspected with the variance inflation factor (highest VIF 4.4 for age). Regression analyses were performed in SPSS, version 26. A post hoc power analysis (power = 1—type II error) was performed in R statistics, package PWR, v1.3–0. The test power was 0.72 to detect a small effect size and 1 to detect a large effect size.

## Results

Overall, the most severe maladaptive personality trait facets (*M* ≥ 1.50) among ED patients were found for the following trait facets (see also Table 1): emotional lability submissiveness, anxiousness, rigid perfectionism, and distractibility. The mean well-being scores were *M* = 2.52 (*SD* = 1.07) for emotional, *M* = 2.55 (*SD* = 0.99) for psychological and *M* = 2.23 (*SD* = 0.99) for social well-being. The mean global EDE-Q score was 4.12 (*SD* = 1.04). A correlation matrix of the variables can be found in the supplements.

The model in step 1, with age, start age ED, BMI kg/m^2^, ED diagnosis, personality disorder, and other psychiatric disorder as independent variables was statistically significant in predicting EWB [*R*^2^ = 0.15, *F* (10, 1154) = 20.94, *p* < 0.001; adjusted *R*^2^ = 0.15], PWB [*R*^2^ = 0.13, *F* (10, 11540) = 16.79, *p* < 0.001; adjusted *R*^2^ = 0.12] and SWB [*R*^2^ = 0.06, *F* (10, 1154) = 7.00, *p* < 0.001; adjusted *R*^2^ = 0.05]. The full model in step 2 with the addition of the personality trait facets led to a statistically significant increase in the explained variance for EWB [change in *R*^2^ = 0.38, *F-*change (25, 1129) = 37.04, *p* < 0.001; adjusted *R*^2^ = 0.52], PWB [change in *R*^2^ = 0.39, *F-*change (25, 1129) = 37.09, *p* < 0.001; adjusted *R*^2^ = 0.51] and SWB [change in *R*^2^ = 0.26, *F-*change (25, 1129) = 17.51, *p* < 0.001; adjusted *R*^2^ = 0.30].

Statistically significant variables associated with well-being in step 2 can be found in Table 2. Anhedonia (*β* = − 0.46, emotional; *β* = − 0.28, psychological; *β* = − 0.23, social), and depression (*β* = − 0.34, emotional; *β* = − 0.32, psychological; *β* = − 0.16, social) were associated with all well-being dimensions over and beyond demographic and illness characteristics. Eccentricity (*β* = 0.07) and submissiveness (*β* = 0.12) were associated with EWB, in addition to EDP severity (*β* = − 0.07). Distractibility (*β* = 0.07), emotional lability (β = -0.08), and manipulativeness (β = 0.08) were associated with PWB, in addition to BN (*β* = − 0.09). Manipulativeness (*β* = 0.08), suspiciousness (*β* = − 0.10), and withdrawal (*β* = − 0.17) were associated with SWB, in addition to having a personality disorder (*β* = − 0.06).

**Table 2:** Results regression analysis: (standardized) beta’s of the independent variables for each dependent variable

	Emotional well-being						Psychological well-being						Social well-being					
	Step 1			Step 2			Step 1			Step 2			Step 1			Step 2		
	B	*SE*	*β*	B	*SE*	*β*	B	*SE*	*β*	B	*SE*	*β*	B	*SE*	*β*	B	*SE*	*β*
Background characteristics																		
Age	0.01	0.01	0.08	0.00	0.01	0.02	.01	.01	.08	0.00	0.01	0.01	0.01	0.01	0.05	0.00	0.01	0.01
Startage ED	− 0.00	0.01	− 0.01	− 0.00	0.01	− 0.02	− .01	.01	− .03	− 0.01	0.01	− 0.05	− 0.01	0.01	− 0.03	− 0.01	0.01	− 0.05
ED duration	− 0.01	0.01	− 0.05	0.00	0.01	− 0.02	− .01	.01	− .10	− 0.01	0.01	− 0.06	− 0.01	0.01	− 0.07	− 0.01	0.01	− 0.06
BMI (kg/m^2^)	0.01	0.01	0.09*	0.01	0.01	0.04	.01	.01	.08	0.01	0.00	0.04	0.00	0.01	− 0.01	0.00	0.00	− 0.02
EDP severity	− 0.26	0.03	− 0.26***	− 0.08	0.02	− 0.07**	− .22	.03	− .23***	− 0.01	0.02	− 0.02	− 0.15	0.03	− 0.16***	0.00	0.03	0.00
AN	− 0.25	0.08	− 0.11**	− 0.09	0.06	− 0.04	− .18	.07	− .09*	− 0.02	0.06	− 0.00	− 0.04	0.08	− 0.02	0.09	0.07	0.04
BN	− 0.08	0.08	− 0.03	− 0.01	0.06	0.00	− .23	.07	− .10**	− 0.17	0.06	− 0.09*	− 0.17	0.08	− 0.07*	− 0.07	0.07	− 0.03
BED	− 0.15	0.12	− 0.04	− 0.01	0.09	0.00	− .15	.11	− .05	− 0.06	0.09	− 0.07	0.00	0.12	0.00	0.09	0.10	0.03
Personality disorder	− 0.31	0.10	− 0.09**	− 0.13	0.07	− 0.04	− .24	.09	− .08**	− 0.10	0.07	− 0.02	− 0.25	0.09	− 0.08**	− 0.17	0.08	− 0.05*
Psychiatric disorder	− 0.40	0.06	− 0.19***	− 0.08	0.05	− 0.04	− .35	.06	− .18***	− 0.05	0.04	− 0.03	− 0.20	0.06	− 0.10**	0.01	0.05	0.00
Personality trait facets																		
Anhedonia				− 0.82	0.07	− 0.46***				− 0.46	0.06	− 0.28***				− 0.38	0.07	− 0.23***
Anxiousness				0.08	0.06	0.05				0.10	0.05	0.07				0.01	0.07	0.01
Attention seeking				− 0.03	0.05	− 0.02				0.06	0.05	0.04				0.02	0.06	0.01
Callousness				0.21	0.13	0.04				0.04	0.12	0.01				− 0.25	0.15	− 0.06
Deceitfulness				− 0.13	0.08	− 0.05				− 0.13	0.08	− 0.06				0.02	0.09	0.01
Depression				− 0.55	0.07	− 0.34***				− 0.47	0.06	− 0.32***				− 0.25	0.07	− 0.16**
Distractibility				− 0.02	0.04	− 0.01				− 0.09	0.04	− 0.07*				0.00	0.05	0.00
Eccentricity				0.12	0.05	0.07*				− 0.03	0.05	− 0.02				− 0.04	0.06	− 0.02
Emotional lability				− 0.04	0.05	− 0.03				− 0.11	0.04	− 0.08*				− 0.04	0.05	− 0.03
Grandiosity				− 0.14	0.08	− 0.05				0.09	0.08	0.03				0.06	0.05	0.03
Hostility				− 0.03	0.06	− 0.02				0.09	0.06	0.05				− 0.06	0.07	− 0.03
Impulsivity				0.00	0.04	0.00				0.06	0.04	0.04				0.04	0.05	− 0.03
Intimacy avoidance				− 0.07	0.04	− 0.05				− 0.02	0.03	− 0.01				0.06	0.04	0.04
Irresponsibility				0.08	0.06	0.04				− 0.09	0.06	− 0.05				− 0.10	0.07	− 0.05
Manipulativeness				0.12	0.07	0.06				0.16	0.06	0.08*				0.15	0.07	0.08*
Percept. dysreg				− 0.11	0.08	− 0.05				− 0.01	0.08	0.00				− 0.07	0.09	− 0.03
Perseveration				0.10	0.06	0.05				0.02	0.06	0.01				0.07	0.07	0.04
Restricted affec				0.09	0.05	0.05				− 0.02	0.05	− 0.01				0.09	0.06	0.05
Rigid perfectionism				− 0.05	0.05	− 0.03				0.00	0.04	0.00				0.00	0.05	0.00
Risk taking				− 0.01	0.06	− 0.01				0.02	0.05	0.01				0.02	0.06	0.01
Separation ins				0.00	0.04	0.00				− 0.04	0.04	− 0.03				− 0.03	0.05	− 0.02
Submissiveness				0.17	0.04	0.12***				− 0.05	0.03	− 0.04				0.04	0.04	0.03
Suspiciousness				− 0.04	0.05	− 0.02				− 0.08	0.05	− 0.05				− 0.17	0.05	− 0.10**
UBE				0.03	0.07	0.01				0.03	0.07	0.01				− 0.08	0.08	− 0.03
Withdrawal				− 0.02	0.06	− 0.01				− 0.08	0.05	− 0.05				− 0.27	0.06	− 0.17***
* R*^2^	.15			.54			.13			.52			.06			.32		
* F*	20.94***			37.12***			16.71***			35.00***			7.00***			15.22***		
Δ*R*^2^	–			.38			–			.39			–			.26		
Δ*F*	–			37.04***			–			37.09***			–			17.51***		

*BMI* body mass index, *EDP severity* eating disorder psychopathology severity, *Psychiatric disorder* other psychiatric disorders, except for personality disorders, *Percept. Dysreg.* perceptual dysregulation, *Restricted affect.* restricted affectivity, *Separation ins. *separation insecurity, *UBE* unusual beliefs and expectations

Emotional, psychological, and social well-being are the dependent variables, * = *p *value < .05, ** = *p* value < .01, *** = *p* value < .001. Step 1 = regression analysis with patient background characteristics, Step 2 = regression analysis, with step 1 variables including the personality trait facets

## Discussion

Decades of research have highlighted the critical role of personality for the experience of well-being in the general population [2]. Studies among ED patients have primarily examined the role of personality in explaining ED pathology [5]. Much less is known about its role for experiencing well-being, while ED patients report lower functioning on well-being compared to the general population. The addition of personality trait facets, above patient background and illness characteristics, led to a statistically significant increase of the explained variance in EWB(38%), PWB (39%), and SWB (26%). Personality trait facets may play a critical role in the experience of well-being among ED patients. Anhedonia and depression were strongly and negatively associated with all three well-being dimensions. These traits are also well linked to ED symptomatology [5]. Personality may function as an underlying mechanism maintaining both psychopathological symptoms and the experience of well-being. It may therefore be critical to focus on strengthening personality trait facets, especially depression and anhedonia, in treatment to promote overall mental health (i.e., low levels of psychopathology and adequate well-being). Farstad and colleagues (2016) also concluded that it is important to capitalize on knowledge about personality in the treatment of EDs, for instance, by tailoring treatments based on personality dimensions. In addition, it may be fruitful to examine the effectiveness of treatments on ED symptom remission and well-being specifically targeting personality functioning, such as dialectical behaviour therapy (DBT) and schema therapy [10].

Several personality trait facets were associated with specific well-being dimensions that may be of interest for clinicians. Emotional lability and distractibility were negatively associated with PWB. Studies have suggested that emotional lability is also associated with EDs, particularly those that involve binge eating [5]. Withdrawal and suspiciousness were associated with lower EWB. Withdrawal is related to avoidance motivation and lower levels of extraversion, which are found to be frequently present in individuals with EDs, as well as suspiciousness among individuals with BN [5]. The review of Farstad and colleagues (2016) suggests that individuals with EDs consistently avoid situations associated with punishment, which may be a pathway to lower societal functioning and SWB.

Further relations were that eccentricity and submissiveness were associated with EWB, and manipulativeness with PWB and SWB. There may be specific pathways to explain these associations, which should be a topic for further investigation. For instance, a person who is anxious for situations with punishment, for instance, for receiving criticism from others, may not only avoid this (withdrawal) but may behave submissive and with that experience adequate levels of EWB as long as they can avoid criticism. Also, for manipulativeness, this may be a way to achieve things in one’s environment, such as getting things their way in treatment or in daily life. Achieving things in the own environment is related to environmental mastery (PWB) [3]. Third-wave behavior therapies, such as acceptance and commitment therapy and compassion focused therapy may be especially effective in promoting mental health because they target these response-focused emotion regulation strategies by fostering acceptance, mindfulness, metacognition, psychological flexibility, and reducing experiential avoidance [10].

Overall, similar associations between personality trait facets and well-being were found in our sample as in the general population [2]. Hostility and callousness were not related to well-being in our sample, in contrary to the general population [2]. Some personality traits are not measured by the PID-5, such as trust, competence, achievement striving, and self-discipline.

### Limitations

It has been suggested that the measurement of stable personality traits may lead to biased results in adolescent samples because they are still in development [1]. In this sample, however, the average age of the patients was 27 years, and the majority of the sample were adult patients. A major limitation is that this is a cross-sectional study meaning that no causal inferences can be made. All patients were females referred for specialized ED treatment, so results may not be generalizable to other ED patients in the community. No information was obtained from patients who did not give consent for this study. It is unknown how this may have affected the results. Also, there may be overlap in the constructs measuring well-being and personality trait facets [2]. Another limitation is that this study did not examine differences between ED types, while it is suggested that different personality trait facets may be linked to specific ED types [5], although we did control for ED type in the analysis. At last, the questionnaires were self-report measures, and results may have been influenced by social desirability as reported by Anglim and colleagues (2020).

## Conclusions and implications

In support of earlier studies in the general population, maladaptive personality trait facets may play a critical role in the experience of well-being among patients with EDs. Clinicians should be aware of potential associations between maladaptive personality traits such as anhedonia and depression with well-being. A focus on these personality traits in treatment may be critical to promote and improve well-being and overall mental health in ED patients.

## What is already known on this subject?

Multiple personality trait facets are well linked with the experience of emotional, psychological, and social well-being in the general population, while much less is known about potential associations in ED patients.

## What do we now know as a result of this study that we did not know before?

Several personality trait facets are moderately or strongly linked with one or more well-being dimensions in patients with EDs. Anhedonia and depression were strongly associated with all well-being dimensions. Personality functioning may be important to focus on in treatment to improve overall mental health.

## Supplementary Information

Below is the link to the electronic supplementary material.

Supplementary file1 (DOCX 35 KB)

## Data Availability

The data that support the findings of this study are available upon reasonable request. The data are not publicly available due to ethical restrictions (e.g., although the data set was anonymized before analysis, new technological procedures may compromise the privacy of the patients).
