# Supplementary material for: Exploring associations between personality trait facets and emotional, psychological and social well-being in eating disorder patients
Source: Eat Weight Disord. 2021 Mar 9;27(1):379–86. doi: 10.1007/s40519-021-01107-6 (PMC8860946; doi:10.1007/s40519-021-01107-6)
Supplement: Supplementary file 1 [file 40519_2021_1107_MOESM1_ESM.docx]

Table 1: correlation matrix

		1	2	3	4	5	6	7	8	9	10	11	12	13	14	15	16	17	18	19	20	21	22	23	24	25	26	27	28	29	30	31	32	33
Emotional well-being	1																																	
Psychological well-being	2	.695**																																
Social well-being	3	.514**	.660**																															
Age	4	.096**	0.044	0.001																														
Startage ED	5	0.026	0.027	0.016	.251**																													
ED duration	6	.061*	0.002	-0.026	.718**	-.301**																												
BMI (kg/m2)	7	.123**	.071*	-0.003	.347**	-.150**	.419**																											
EDP severity	8	-.277**	-.257**	-.182**	-.079**	-0.049	-0.026	0.054																										
Anhedonia	9	-.674**	-.632**	-.476**	-.100**	-0.047	-0.048	-.093**	.227**																									
Anxiousness	10	-.392**	-.419**	-.336**	-.230**	-.081**	-.142**	-.112**	.270**	.489**																								
Attention seeking	11	.077**	.101**	0.054	-0.006	-0.003	0.001	.091**	-0.014	-.086**	.119**																							
Callousness	12	-.058*	-0.048	-.134**	-0.027	-0.006	-0.031	0.012	-0.036	.181**	.098**	.265**																						
Deceitfulness	13	-.125**	-.137**	-.129**	-.121**	-.081**	-.058*	.063*	.069*	.213**	.266**	.435**	.526**																					
Depression	14	-.648**	-.660**	-.468**	-.150**	-.076**	-.078**	-.110**	.367**	.748**	.652**	-0.048	.079**	.199**																				
Distractibility	15	-.320**	-.389**	-.277**	-.099**	-.107**	-0.012	0.028	.240**	.441**	.448**	.126**	.143**	.262**	.469**																			
Eccentricity	16	-.269**	-.323**	-.293**	-.194**	-.090**	-.116**	-.110**	.155**	.383**	.488**	.190**	.284**	.388**	.464**	.438**																		
Emotional lability	17	-.252**	-.303**	-.252**	-.093**	-.065*	-0.035	0.041	.200**	.259**	.528**	.166**	.063*	.186**	.421**	.339**	.441**																	
Grandiosity	18	.074*	.140**	0.041	-0.027	0.022	-0.040	0.026	-.134**	-.069*	0.055	.439**	.358**	.335**	-.142**	0.030	.231**	.129**																
Hostility	19	-.199**	-.165**	-.232**	-0.026	-0.017	-0.012	0.027	.105**	.309**	.307**	.271**	.523**	.450**	.237**	.316**	.397**	.339**	.272**															
Impulsivity	20	-0.049	-.081**	-.082**	-.058*	-0.044	-0.014	.120**	.120**	.064*	.152**	.307**	.193**	.295**	.141**	.419**	.379**	.255**	.129**	.293**														
Intimacy avoidance	21	-.342**	-.320**	-.211**	-.166**	-0.050	-.126**	-.153**	.109**	.410**	.279**	-0.030	.114**	.168**	.393**	.269**	.281**	.071*	0.042	.124**	0.036													
Irresponsibility	22	-.156**	-.240**	-.220**	-.132**	-.092**	-.071*	.065*	.108**	.261**	.350**	.282**	.334**	.495**	.288**	.497**	.454**	.250**	.203**	.340**	.467**	.183**												
Manipulativeness	23	0.038	.081**	0.030	-0.044	-0.029	-0.020	0.027	-0.014	0.031	.106**	.503**	.439**	.639**	-0.026	.136**	.229**	.087**	.463**	.391**	.188**	0.046	.287**											
Perceptual dysregulation	24	-.274**	-.301**	-.273**	-.186**	-.093**	-.114**	-.076**	.193**	.326**	.492**	.167**	.210**	.338**	.438**	.449**	.648**	.436**	.219**	.268**	.302**	.298**	.440**	.202**										
Perseveration	25	-.321**	-.365**	-.277**	-.120**	-.075**	-0.033	-.098**	.168**	.463**	.588**	.201**	.166**	.310**	.502**	.528**	.567**	.429**	.168**	.340**	.212**	.303**	.367**	.202**	.547**									
Restricted affectivity	26	-.294**	-.294**	-.205**	-.157**	-0.021	-.125**	-.149**	.095**	.489**	.165**	-0.034	.240**	.257**	.350**	.246**	.272**	-.149**	0.048	.191**	.077**	.413**	.203**	.155**	.273**	.252**								
Rigid perfectionism	27	-.249**	-.228**	-.179**	-.142**	0.020	-.111**	-.234**	.175**	.319**	.442**	.057*	.086**	.134**	.336**	.184**	.336**	.297**	.142**	.259**	-0.049	.184**	0.024	.148**	.322**	.601**	.186**							
Risk taking	28	.085**	.114**	.089**	-.168**	-0.018	-.141**	-.154**	-0.020	-.149**	-.143**	.237**	.169**	.168**	-.091**	.088**	.199**	-.070*	.186**	.078**	.393**	0.030	.205**	.174**	.168**	-0.018	.112**	-.084**						
Separation insecurity	29	-.198**	-.244**	-.168**	-.198**	-.058*	-.144**	-0.053	.179**	.215**	.463**	.240**	0.053	.244**	.385**	.261**	.248**	.420**	0.053	.206**	.169**	0.023	.239**	.142**	.264**	.320**	0.045	.229**	-0.052					
Submissiveness	30	-.206**	-.336**	-.178**	-.130**	-0.028	-.074*	-.088**	.183**	.346**	.412**	.064*	-0.044	.156**	.449**	.251**	.231**	.247**	-.077**	0.012	0.045	.173**	.160**	0.011	.241**	.354**	.178**	.311**	-.172**	.317**				
Suspiciousness	31	-.305**	-.350**	-.341**	-.099**	-0.047	-0.056	-0.012	.213**	.368**	.458**	.065*	.202**	.246**	.460**	.296**	.428**	.316**	.095**	.361**	.184**	.289**	.269**	.073*	.436**	.365**	.254**	.281**	0.036	.308**	.268**			
UBE	32	-.113**	-.126**	-.182**	-0.025	-.097**	0.029	0.022	.077**	.133**	.319**	.147**	.170**	.279**	.193**	.238**	.502**	.309**	.329**	.249**	.185**	.191**	.284**	.241**	.640**	.348**	.126**	.238**	.136**	.117**	.059*	.352**		
Withdrawal	33	-.450**	-.482**	-.436**	-.066*	-.073*	-0.017	-.067*	.212**	.623**	.421**	-.143**	.202**	.244**	.560**	.347**	.441**	.248**	0.035	.306**	.060*	.476**	.292**	0.054	.395**	.413**	.491**	.322**	-.131**	.092**	.269**	.423**	.282**	

**. Correlation is significant at the 0.01 level (2-tailed).

*. Correlation is significant at the 0.05 level (2-tailed).
